# The Influence of the Non-Pathogenic *Fusarium oxysporum* Fo47 Strain on Flax Resistance to Pathogens

**DOI:** 10.3390/ijms26094396

**Published:** 2025-05-06

**Authors:** Justyna Liszka, Lucyna Dymińska, Wojciech Łaba, Magdalena Wróbel-Kwiatkowska

**Affiliations:** 1 Department of Biotechnology and Food Microbiology, Faculty of Biotechnology and Food Science, Wrocław University of Environmental and Life Sciences, 51-630 Wrocław, Poland; 121244@student.upwr.edu.pl (J.L.); wojciech.laba@upwr.edu.pl (W.Ł.); 2 Department of Bioorganic Chemistry, Wroclaw University of Economics and Business, 53-345 Wrocław, Poland; lucyna.dyminska@ue.wroc.pl

**Keywords:** *Linum usitatissimum* L., Fo47, FTIR, flavonoids, phenolic compounds, biocontrol

## Abstract

Flax (*Linum usitatissimum* L.) is a plant of high economic and practical importance valued for its fiber and oil, which have diverse applications in industries such as textiles, food, pharmaceuticals, and construction. Fungal pathogens of the genus *Fusarium*, however, pose one of the most serious threats to flax cultivation. They are responsible for a number of disease manifestations, notably *Fusarium* wilt and root rot. In the case of fusariosis, there is a lack of plant protection products, and often the only effective approach is to use resistant flax cultivars or to discontinue cultivation for several years. Currently, much attention is paid to biological methods of plant protection, which do not exert a negative influence on the environment or human health and are important for sustainable agriculture. The aim of the present study was to assess the potential of the non-pathogenic endophytic fungal strain *Fusarium oxysporum* Fo47 in protecting plants against pathogenic fungi. The results showed that pretreatment of flax plants with Fo47 increased resistance of plants to all tested fungi (*F. oxysporum*, *Fusarium culmorum*, *Rhizoctonia solani*). Fo47 was the most effective for protection against *F. culmorum* for the Jan flax cultivar and *R. solani* for the Bukoz cultivar. Pretreatment with Fo47 of flax plants inoculated with *F. culmorum* caused an increase in the level of secondary metabolites involved in plant resistance (phenolics) and photosynthetic pigments (chlorophyll a and b) compared to plants treated only with the pathogenic fungal strain. Fourier transform infrared spectroscopy revealed structural changes in the polymers of cell walls. The highest intensities of vibrations characteristic of lignin and pectin were observed for flax treated with Fo47 and infected with *F. culmorum*, suggesting the highest level of these polymers, higher than in plants treated only with pathogenic fungi. Thus, it can be concluded that application of the non-pathogenic strain strengthened the immune response of flax plants. These results highlight the strong potential of the non-pathogenic strain as a biological control agent, especially for *Fusarium* infection in flax.

## 1. Introduction

Flax (*Linum usitatissimum* L.) has been cultivated for over 6000 years, making it one of the oldest-known cultivated crops [1]. Currently, it is grown in more than 50 countries, with Canada, India, Russia, Kazakhstan, and China being the largest producers.

Flax is a valuable crop due to its seeds and fibers. The literature suggests that flax can be successfully cultivated in various regions of the European Union, with a potentially low environmental impact [2]. Being a functional food, the seeds are fortified with lignans, alpha-linolenic acid (ALA), and dietary fiber. Their consumption is associated with lower risks of cardiovascular diseases, atherosclerosis, diabetes, cancer, and autoimmune disorders [3]. Long fibers of flax are used in textiles, while short fibers are applied in paper, insulation materials, and biocomposites. Byproducts from flax processing can serve as an energy source, and flaxseed meal, rich in antioxidants, is a food supplement. The seeds and oil can be also used in the pharmaceutical and cosmetic industries [4]. Diseases, e.g., fusariosis caused by fungi, pose a significant threat to seed and fiber quality, emphasizing the importance of effective management strategies [5].

Fungi of the *Fusarium* genus are major plant pathogens responsible for severe crop losses worldwide. In flax (*Linum usitatissimum* L.), they cause diseases such as vascular wilt and root rot, which can lead to as much as 80–100% loss of yield if favorable conditions for pathogen development occur [6]. *Fusarium* belongs to the soil-borne pathogens, and thus disease-favoring conditions are wet environments, acidic pH of soil (soil suppressive of *Fusarium* has pH 7.9), and no microorganism antagonists [5]. The pathogenicity of *Fusarium* strains, in this case, involves colonization of the roots and vascular tissues of the plant, thereby disrupting water and nutrient transport, leading to the death of the plant. Mycotoxins such as fumonisins, zearalenone, and trichothecenes also pose a great danger to crop quality and safety. There is a lack of plant protection products for limiting fungi, especially as there are fungicide-resistant strains (belong to the *Fusarium* genus), which are viable in soils for long periods (up to seven years). This has led to research seeking alternative methods for inhibiting pathogen growth and crop protection. *Fusarium oxysporum* Fo47, a non-pathogenic strain, was first isolated from suppressive soils associated with vascular diseases in which its activity contributed to limiting pathogen development. Studies have shown that the reintroduction of Fo47 into sterilized soil restores its suppressive properties [7]. The biocontrol activity of *F. oxysporum* Fo47 has been confirmed in several plant species, including tomato (*Solanum lycopersicum* L.) [8,9] pepper (*Capsicum annuum* L.) [10,11], pea (*Pisum sativum* L.) [12], and potato (*Solanum tuberosum* L.) [13].

The colonization process begins with spore germination or hyphal growth stimulated by sugars secreted by plant roots. Both endophytic and pathogenic strains initially colonize the root surface and subsequently penetrate plant tissues through epidermal damage, cracks, or lateral root emergence points [14]. Hyphae migrate through the root apoplast to the vascular stele, with intracellular growth being more frequently observed in non-pathogenic strains [15]. The *F. oxysporum* Fo47 strain restricts colonization to the epidermal layer and outer cortical cells using specialized structures known as penetration pegs, which facilitate tissue penetration. During colonization, Fo47 induces plant defense mechanisms, including the deposition of osmiophilic material, the formation of fibrillar networks, and the accumulation of granules in intercellular spaces [16]. Research on the impact of Fo47 on the enhanced synthesis of secondary metabolites remains relatively limited. The previous study demonstrated that *F. oxysporum* Fo47 stimulates structural changes in the cell walls of flax callus cultures, including an increase in cellulose crystallinity and an almost twofold rise in polyphenol and flavonoid concentrations [17]. These findings, as well as present results (obtained on whole plants), suggest that Fo47 not only induces the production of secondary metabolites but also modifies the chemical composition of the plant cell wall, making it a potential agent for biological crop protection.

## 2. Results and Discussion

### 2.1. Antagonistic Effect of Non-Pathogenic Strain F. oxysporum Fo47 on Pathogenic F. oxysporum

**Figure 1:**
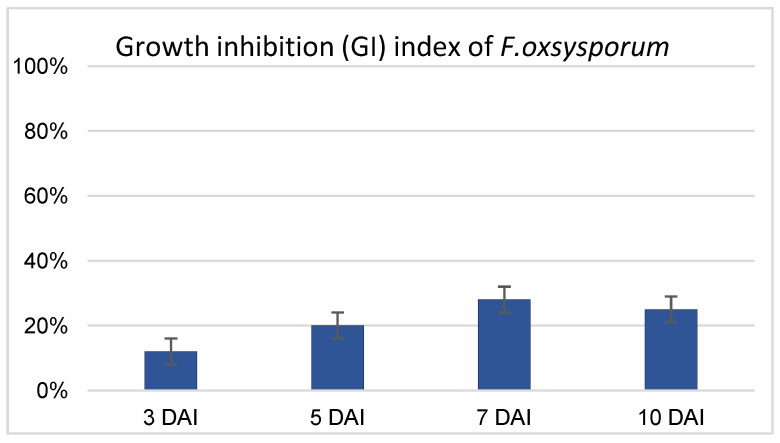
Determination of biocontrol properties of non-pathogenic strain of *F. oxysporum* Fo47 against pathogenic strain of *F. oxysporum*. Growth inhibition (GI) index assessed as described in Materials and Methods section. The analyses were performed at 3, 5, 7, and 10 days after inoculation. Values represent the means ± SE of three samples.

Based on data obtained in previous work (Wróbel-Kwiatkowska et al., 2024) [17], which confirmed the antagonistic activity of non-pathogenic *F. oxysporum* strain Fo47 against the pathogenic fungi *F. culmorum* and *Rhizoctonia solani*, the activity of Fo47 against *F. oxysporum* was tested in the present study (Figure 1). *F. oxysporum* is one of the most common flax pathogens, and the protective role of the non-pathogenic Fo47 strain against this strain was determined in the dual-culture assay following the protocol described in the Materials and Methods section. The results indicate that the optimal effect was observed after 7 days of common culture of pathogenic and nonpathogenic *F. oxysporum* strains, with growth inhibition (GI) of 28%. At 10 days after inoculation (DAI), GI had decreased to 25% (Figure 1). The antagonistic effect was higher than for *F. culmorum*, but weaker than that observed for *R. solani*, for which maxima of 22% and 45% growth inhibition, respectively, were determined when the Fo47 strain was used (Wróbel-Kwiatkowska et al., 2024) [17].

### 2.2. Determination of Flax Resistance to Fungi and Impact of Non-Pathogenic Strain Fo47

**Figure 2:**
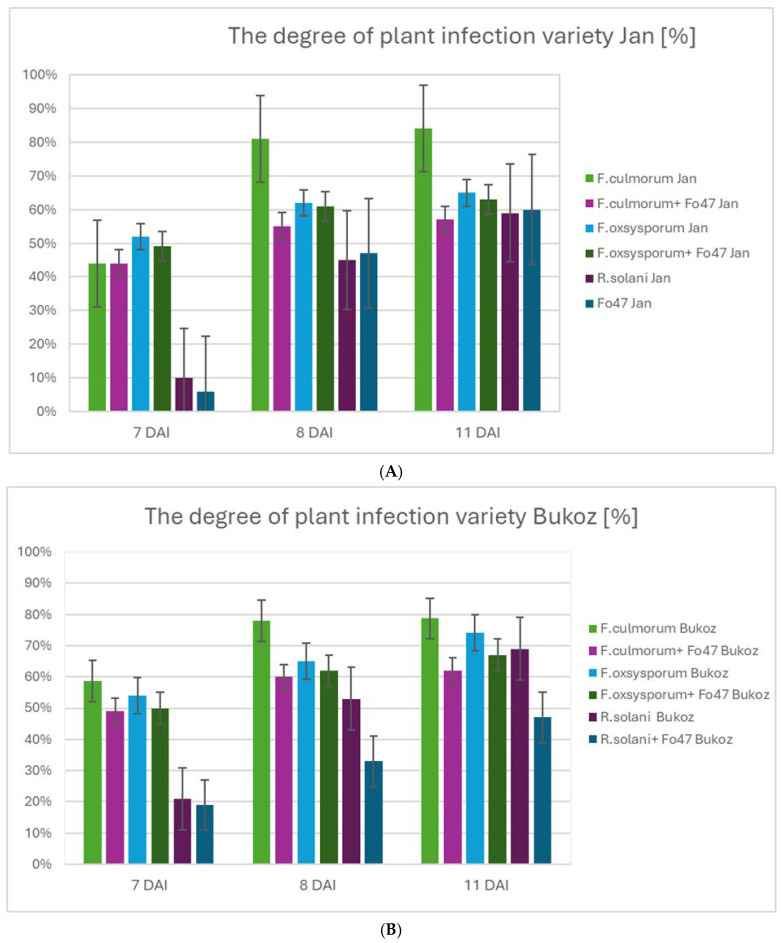
The degree of plant infection caused by pathogenic strains in flax grown in in vitro cultures. (**A**) Degree of infection estimated for flax of the Jan cultivar. (**B**) Degree of infection estimated for flax of the Bukoz cultivar. 30 plants were analyzed for each experimental variant. Chi-squared tests revealed significant differences in infection level distributions between experimental groups across assessment days for both cultivars (Jan cultivar—DAI 7: χ^2^ = 65.60, *p* < 0.0001; DAI 8: χ^2^ = 40.32, *p* = 0.0004; DAI 11: χ^2^ = 19.20, *p* = 0.2046; Bukoz cultivar—DAI 7: χ^2^ = 39.44, *p* = 0.0006; DAI 8: χ^2^ = 42.68, *p* = 0.0002; DAI 11: χ^2^ = 36.53, *p* = 0.0015).

The influence of the non-pathogenic fungus *F. oxysporum* Fo47 on flax plants infected with the pathogenic fungus strains *F. culmorum*, *F. oxysporum*, and *R. solani* was determined for two Polish flax cultivars: Jan and Bukoz (Figure 2). An important factor contributing to reduced flax yields is the prevalence of diseases caused by fungi, especially from the *Fusarium* genus. Recently, due to climate change, the importance of the necrotrophic fungus *R. solani* in flax diseases has also increased [18]. Thus, it can be stated that *Fusarium* and *R. solani* belong to ubiquitous soil-borne fungi, important from an economic point of view, because they remain the main reasons for flax diseases, resulting in a severe decrease in plant yield. Analysis of the infection index in in vitro flax cultures revealed significant differences depending on the pathogen type and the presence of the *F. oxysporum* Fo47 strain.

For the flax plants of the two tested cultivars, the degree of infection was the lowest for *R. solani* at 7 DAI, and in the case of Fo47, the degree of infection decreased, especially for the Bukoz cultivar after 8 and 11 days of inoculation (Figure 2B).

The highest infection rate observed for *F. culmorum*, reaching 44% in the Jan cultivar was noticed at day 7 and increased to 81% at day 8 and 84% at day 11. The presence of Fo47 significantly reduced the infection rate, reducing it to 55% (day 8) and 57% (day 11). Compared to *F. culmorum*, the infection of the Jan cultivar caused by *F. oxysporum* was slightly lower, with a maximum of 65% on day 11. Co-inoculation with Fo47 reduced the infection rate to 63%. For *R. solani* and the Jan cultivar, no protective effect was observed after 11 days of common culture of pathogenic and non-pathogenic fungi (Figure S1).

In the case of infection with *F. culmorum* and the Bukoz cultivar, the infection severity reached 58% (7 DAI), increasing to 77% on day 8 and 78% on day 11. The introduction of Fo47 effectively suppressed infection progression, reducing it to 60% (day 8) and 62% (day 11). Infections caused by *F. oxysporum* were lower than those of *F. culmorum*, with a peak of 74% on day 11, which was further reduced to 67% in the presence of Fo47. The lowest infection levels were observed for *R. solani*, with a 69% infection rate on day 11, which was reduced to 47% after co-inoculation with Fo47 (Figure 2A and Figure S2).

Observed changes in the resistance level among the cultivars are in accordance with literature data, which indicate that the pathogenicity of fungi depends on the flax genotype/cultivar [6].

Due to the high efficacy of Fo47 against the pathogen *F. culmorum* and the fact that fungi from the *Fusarium* genus are major flax pathogens with economic importance, this pathogen was selected for further analysis in the context of its impact on secondary metabolite content and plant cell wall structure and composition.

It should be pointed out that nowadays, more than 130 antifungal secondary metabolites isolated from different endophytes have been determined [19], among them being cytochalasin alkaloids, polyketides, terpenoids, and other compounds, e.g., piliformic acid, cordycepsidone A, etc. Some of these compounds exhibit desirable bioactive properties (antimicrobial, antimalarial, neuroprotective, and anticancer) and enzymatic activities (cellulases, proteases, lipases etc.), and some of them are completely new metabolites, e.g., colletotrichine A from *Colletotrichum gloeosporioides* [20]. Antifungal compounds synthesized by endophytic strains, used in this study, were not assessed, but will be evaluated in the near future.

### 2.3. Determination of the Pigment Content in Flax in In Vitro Cultures

**Figure 3:**
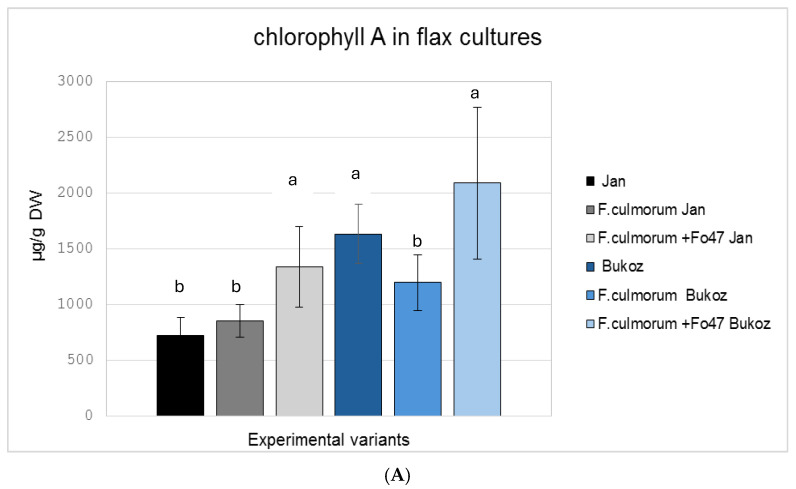

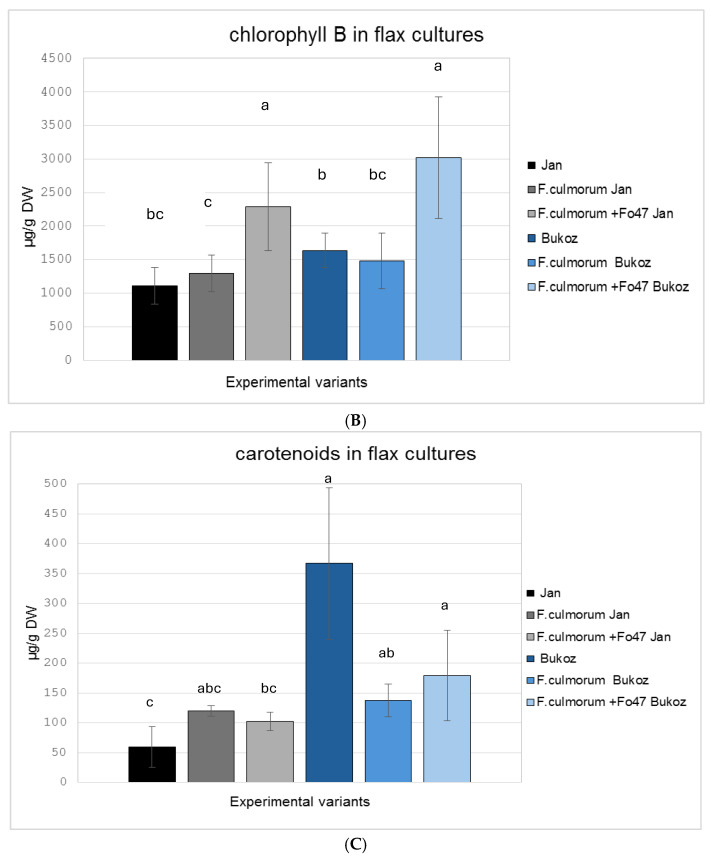
The effect of fungal infection in flax grown in tissue cultures on the content of photosynthetically active pigments: (**A**) chlorophyll a, (**B**) chlorophyll b, and (**C**) carotenoids. Values represent the means ± SD of six samples.

The analysis of content of photosynthetically active pigments (chlorophyll a, chlorophyll b, and carotenoids) revealed differences depending on the experimental conditions (Figure 3).

Under co-infection conditions with *F. oxysporum* Fo47 and *F. culmorum*, increased accumulation of pigments was observed, leading to elevated of chlorophyll a and chlorophyll b content for both tested flax cultivars (Figure 3A,B). In the Bukoz cultivar, infection with the pathogenic strain *F. culmorum* alone caused pigment degradation, i.e., lower levels of chlorophyll a (this change was statistically significant) and b, as well as carotenoids, which may indicate a response to stress, caused by a fungal attack. For carotenoids measured in the Jan cultivar, a slight increase was detected after treatment with the pathogenic strain (*F. culmorum*) and non-pathogenic Fo47 strain. This amount was, however, lower than for the uninfected control Bukoz plants. For the Bukoz cultivar, infection with either the pathogenic or non-pathogenic strain caused a decrease in carotenoid levels (Figure 3C).

The observed increase in chlorophyll levels in infected plants could be a compensatory mechanism or a result of metabolic changes associated with stress. Notably, the protective role of Fo47 is evident, as it not only limits pigment degradation but also stimulates their biosynthesis. Similar results to these obtained for the Bukoz cultivar were observed for two maize lines infected with *Fusarium verticillioides*, for which infection caused damage of chlorophyll in both maize lines and a reduction in carotenoids for one tested maize line [21]. Thus, different cultivars and even lines can show various responses to the same agent.

### 2.4. Analysis of Total Phenolic Content (TPC) and Total Flavonoid Content (TFC) in Tested Flax Plants

**Figure 4:**
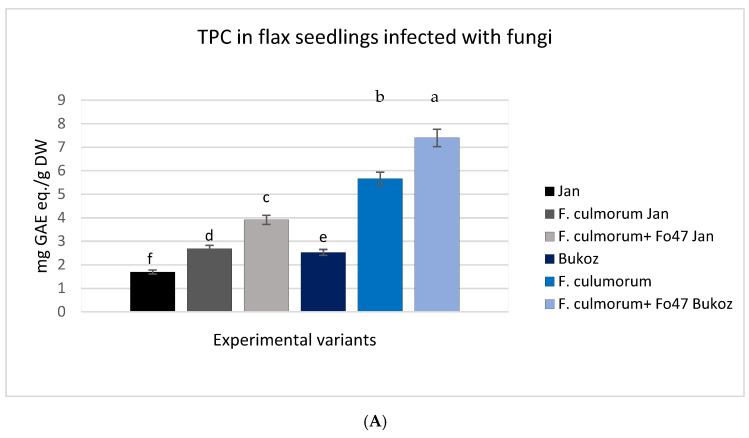

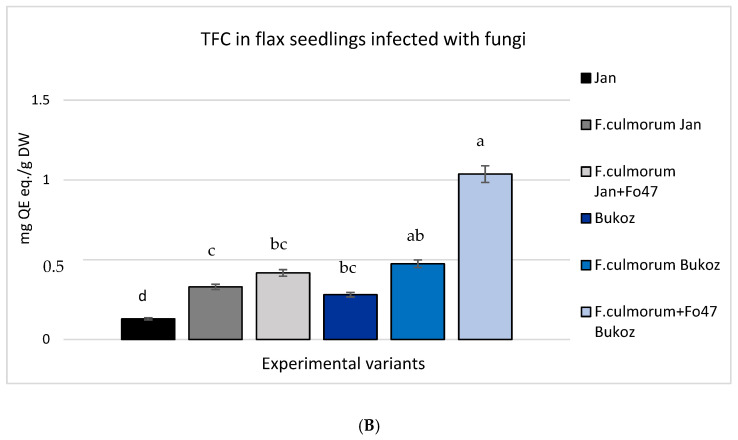
The effect of fungal infection in flax seedlings on the accumulation of bioactive compounds. (**A**) TPC (total phenolic content) and (**B**) TFC (total flavonoid content) were measured as described in the Materials and Methods section. Values represent the means ± SD of three to six samples analyzed per each experimental variant.

Endophytic fungi influence the concentration of phenolic compounds and flavonoids in plants by stimulating metabolic pathways, such as the activation of genes associated with the biosynthesis of phenylpropanoids, flavonols, siderophores, and flavonoids, as well as the induction of systemic resistance. This results in increased production of phenolic compounds and flavonoids as defense mechanisms in plants [22]. Therefore, in the conducted cultures, the concentration of polyphenolic compounds and flavonoids was determined and compared with a control group without the addition of fungal inoculum. The highest concentrations of phenolic compounds and flavonoids were determined in the flax plants infected with both the pathogenic strain and non-pathogenic Fo47 (Figure 4A,B). This result was observed for both tested flax cultivars. However, the largest amounts of phenolics and flavonoids were noted for the Bukoz cultivar treated with both fungi, and the changes were statistically significant. Treatment with Fo47 and infection with the pathogenic strain caused a 2.5-fold increase in TPC and 3.5-fold increase in TFC in the Bukoz cultivar (Figure 4A,B). In each analyzed culture, the addition of *F. oxysporum* Fo47 led to an increase in the content of these secondary metabolites, highlighting its significant role in stimulating resistance to fungi. It has been reported that polyphenols exhibit antimicrobial activities, including antifungal properties [23].

In another study conducted by Veloso et al. (2016) [11], inoculation with Fo47 increased the concentration of caffeic acid in roots within 48 h post-inoculation. The effect of Fo47 on secondary metabolites in pea (*Pisum sativum* L.) roots was evident through the induction of a protective amorphous substance in host cells that was rich in phenolic compounds [12]. Increased levels of polyphenols were also observed after inoculating the flax suspension cultures with the Fo47 strain compared to non-treated control cultures [17].

Flavonoids are also known antifungal molecules [24]. Therefore, in pathogen attacks, flavonoid biosynthesis is activated, highlighting their role in plant resistance. All of these data indicate the protective role of non-pathogenic fungi and the potential mechanism of their action by activation of plant secondary metabolites. Previous results derived from flax suspension cultures also showed that elicitation of cultures with Fo47 strain caused flavonoid accumulation [17]. It should be pointed out that observed changes are in accordance with published data, which showed that endophytes impact the secondary metabolism in plants via changes in gene expression involved in crucial metabolic pathways that provide phenolic compounds [20]. The HMGR (3-hydroxy-3-methylglutaryl–CoA reductase) pathway is regulated by endogenous and exogenous agents [25], among them endophytic fungi. This pathway also provides carotenoids, and thus observed changes in carotenoid levels may be the result of the non-pathogenic strain.

### 2.5. Spectroscopic Analysis of Cell Wall Polymers in Studied Flax Plants

Because analysis of the transcriptome of *Fusarium*-resistant and -susceptible flax plants infected with this pathogen revealed, among other things, changes in expression of genes connected with cell wall organization [6], the aim of this study was to analyze cell wall components in flax protected with a non-pathogenic strain of *Fusarium* (Fo47) and infected with the pathogen *F. culmorum*.

**Figure 5:**
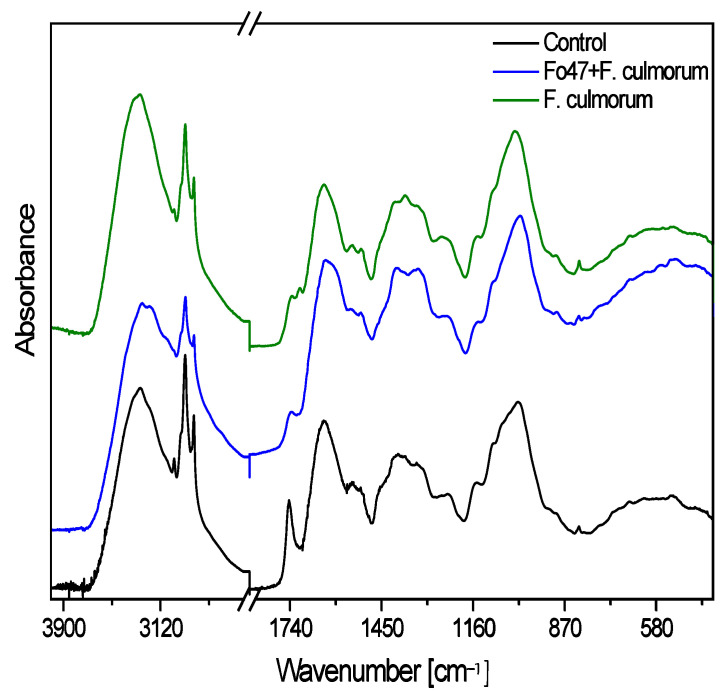
FTIR spectra of the studied samples. The flax plants were infected with *F. culmorum* or *F. culmorum* and simultaneously treated with non-pathogenic strain Fo47. Flax plants not infected with any fungi were used as a control.

The general shape of the measured FTIR spectra of flax fibers is typical of the cellulose spectrum, enriched with lignin and pectin bands (Figure 5). Table 1 presents the dependence of the integral intensities of characteristic cellulose bands for the tested samples. The data include five specific wavenumbers, each corresponding to particular vibrational assignments associated with cellulose structure [26,27,28]. Overall, the variations in intensity suggest structural differences in the cellulose among the samples, with the sample after infection with *F. culmorum* generally showing higher intensity values compared to the untreated control plants and those treated with both pathogenic and non-pathogenic strains (Fo47 + Fc) in most cases.

In the FTIR spectra, the broad absorption bands observed in the range 3700–3000 cm^−^^1^ are associated with hydroxyl groups involved in hydrogen bonding (both intramolecular and intermolecular) [29,30,31,32]. The broad contour of the control sample spectrum was deconvoluted into seven Lorentzian components (3530, 3424, 3390, 3290, 3185, 3070, and 3010 cm^−^^1^), while the spectra of the flax treated with Fo47 and *F. culmorum* contain six components (3530, 3370, 3280, 3180, 3060, and 3024 cm^−^^1^) and the plants infected only with *F. culmorum* contain five components (3527, 3419, 3284, 3187, and 3010 cm^−^^1^). The component at approximately 3530 cm^−^^1^ corresponds to the vibration of free hydroxyl groups, while the other components are associated with the vibrations of inter- and intramolecular hydrogen bonds in cellulose. In the control sample, a greater diversity of inter- and intramolecular hydrogen bonding interactions within cellulose chains was observed compared to samples treated with both fungi.

The crystallinity index (Icr) of cellulose was calculated as the integral intensity ratio of the 1372 cm^−^^1^ and 2920 cm^−^^1^ IR bands [33]. For the studied samples, the calculated crystallinity indexes took the following order: control > sample treated with Fo47 + *F. culmorum* > plants infected with *F. culmorum*. A similar effect was observed for the callus suspension cultures, for which treatment with the non-pathogenic strain of *F. oxysporum* Fo47 caused an increase in the measured Icr index of cellulose (Wróbel-Kwiatkowska et al., 2024) [17].

The bands in the FTIR spectrum at 1545 cm^−^^1^, 1508 cm^−^^1^, 1336 cm^−^^1^, and 826 cm^−^^1^ are typically associated with the characteristic vibrations of the aromatic ring and functional groups present in lignin [34]. The data presented in Table 2 indicate that the integral intensities of the selected characteristic bands for lignin show a dependence in the following samples: control (C), plants infected with *F. culmorum* (Fc), and flax after treatment with Fo47 and inoculated with *F. culmorum* (Fo47 + Fc). In all cases, the intensity values increase in the order C ≤ Fc < Fo47 + Fc, suggesting that the last sample exhibits the highest intensity for these characteristic vibrations. This trend implies structural or compositional differences among the samples, with treatment with Fo47 and *F. culmorum* likely having a higher concentration of aromatic and conjugated functional groups of lignin compared to the other samples. Lignins are chemically polymers of coumaric, coniferyl, and sinapic alcohols, which belong to phenolic compounds. In fact, biochemical analysis (Figure 4) showed the greatest increase in the content of phenolic compounds in plants infected with the pathogen (*F. culmorum*) and simultaneously treated with non-pathogenic *F. oxysporum* Fo47, which is in agreement with data obtained by infrared spectroscopy. Thus, it can be concluded that common treatment with the non-pathogenic strain Fo47 and pathogenic *F. culmorum* remains the main reason for the increased lignin.

Table 3 shows the dependence of the integral intensities of selected characteristic bands for pectin in all tested samples. The analysis focuses on the vibrations of ester carbonyl groups and free or ionized carboxyl groups, which are crucial for understanding the structure and modifications of pectin. In the FTIR spectrum of pectins, the bands at 1740 cm^−^^1^ and 1705 cm^−^^1^ are typically associated with the vibrations of the carbonyl (C=O) group in esterified forms of pectin. The band at 1740 cm^−^^1^ corresponds to the esterified carbonyl groups (C=O) in the methyl-esterified galacturonic acid (COOCH_3_). The band at 1705 cm^−^^1^ can also be attributed to the carbonyl stretching in esterified pectin, but it appears at a slightly lower frequency compared to the 1740 cm^−^^1^ band, often reflecting the presence of acetyl esters or a slightly different environment for ester groups. The shift to a lower wavenumber can also result from hydrogen bonding or other structural factors within the pectin. At 1740 cm^−^^1^, the intensity relationship follows the order control ≈ Fc < Fo47 + Fc, indicating that the sample (after pretreatment with Fo47 and infected with *F. culmorum*) has the strongest signal, suggesting a higher presence of esterified carbonyl groups. A similar trend is observed at 1705 cm^−^^1^ (Fc ≤ C < Fo47 + Fc), reinforcing the observation that sample Fo47 + Fc has the highest concentration of ester groups of pectin. The bands at 1647 cm^−^^1^ and 1600 cm^−^^1^ correspond to the asymmetric and symmetric stretching vibrations of free or ionized carboxyl groups (ν_as_(COO^−^) and ν_s_(COO^−^), respectively) in the pectin structure, particularly in the region related to the ionization or dissociation of carboxyl groups. At 1647 cm^−^^1^, the intensity relationship C ≤ Fc < Fo47 + Fc suggests an increasing presence of ionized carboxyl groups from the untreated control flax plants (C) to flax pretreated with Fo47 and inoculated with *F. culmorum*. A similar trend is noted at 1600 cm^−^^1^ (Fc ≤ C < Fo47 + Fc), further confirming the enhanced presence of free or ionized carboxyl groups in the last sample. Overall, the analysis indicates that pretreatment of flax with the non-pathogenic strain Fo47 and infection with the pathogenic fungus *F. culmorum* resulted in the highest intensity for both ester- and carboxyl-related bands, suggesting the highest concentration of esterified and ionized pectin compared to the other samples. Notably, inoculation of flax plants or callus suspension cultures with only the non-pathogenic fungal strain Fo47 did not cause an increase in pectin synthesis [17,35]. The observed effect is a response of plants to the pathogenic strain (*F. culmorum*), and this effect is intensified when a non-pathogenic strain is used.

Thus, it can be assumed that common application of pathogenic and non-pathogenic fungi affects the structure of plant cell walls by increasing the amount of pectin and lignin. The plant cell wall polymers, especially lignin, play an important role in the response of plants to pathogens [36,37]. The data confirm the view that the plant cell wall is an active structure, which is subjected to remodeling depending on plant growth conditions [38]. Pathogen infection affects the quantity of plant cell wall polymers as well as the structure of the cell wall, as evidenced by the increased crystallinity index of cellulose for infected flax plants. In each experiment, application of the non-pathogenic strain and then inoculation with pathogenic fungi caused an improvement in the plant response to pathogen attack (higher lignin and pectin content, increased crystallinity of cellulose) compared to the plants infected only with the pathogenic *F. culmorum* strain. Thus, these data suggest that the non-pathogenic strain may have high potential as a protective agent, and its application is correlated with higher plant resistance.

## 3. Materials and Methods

### 3.1. Plant Material

Two Polish cultivars of flax—the fibrous Jan cultivar and the oily Bukoz cultivar—were used in the present study. Seeds of these cultivars were obtained from the Institute of Natural Fibers and Medicinal Plants in Poznań, Poland. The flax seeds were sterilized in a 50% plant preservative mixture (PPM™, Plant Cell Technology, WA, USA) for 10 min and then placed on sterilized vermiculite (8 g) supplemented with 50 mL of liquid MS medium (Murashige and Skoog medium) in sterile glass jars. The MS (Murashige and Skoog basal medium, M5519) was obtained from Merck, Darmstadt, Germany. After the seeds germinated (after about 14 days of dark incubation), the seedlings were cultivated under a photoperiod of 10 h light/14 h dark at a temperature of 21 ± 2 °C. Young, healthy seedlings were selected for the experiment.

The jars containing seedlings were divided into two groups: one group was infected only with the pathogenic fungus, while the other was pre-infected with the non-pathogenic *F. oxysporum* strain Fo47, and at 2 DAI, the plants were exposed to the pathogen. The plants were grown for a period of 11 days. Flax plants not exposed to any microorganisms served as the control.

After 11 days of cultivation, the plants were subjected to lyophilization using a LABCONCO freeze-dryer (Labconco, MO, USA).

### 3.2. Fungal Material

The pathogenic strains *F. oxysporum* MYA-1201 and *F. culmorum* DSM 1094 are part of the collection of the Department of Biotechnology and Food Microbiology, Wrocław University of Environmental and Life Sciences. The non-pathogenic *F. oxysporum* strain (ATCC number MYA-1198, Fo47) was obtained from the Department of Genetic Biochemistry, University of Wrocław. *R. solani* was obtained from the Department of Plant Protection, Wrocław University of Environmental and Life Sciences.

### 3.3. Conditions of Fungal Cultures

All fungi used in the current study were inoculated on PDA (potato dextrose agar) and incubated for 10 days at 25 °C. Subsequently, spores were harvested from the culture plates using a 0.1% Tween solution. The suspensions were standardized to achieve a final density of 10⁶ spores/mL.

### 3.4. Determination of Biocontrol Activity of Fo47 Against F. oxysporum

The antagonistic ability of *F. oxysporum* Fo47 against pathogenic *F. oxysporum* was estimated by the dual-culture test described by Wróbel-Kwiatkowska et al., 2024 [17]. The growth inhibition index was calculated at 3, 5, 7, and 10 DAI.

### 3.5. Flax–Fungi Bioassay Determining the Resistance of Tested Flax Plants to Fungal Diseases

All the experiments were performed in sterile tissue cultures of flax plants. Three-week-old seedlings of flax grown in sterile vermiculite were inoculated by pathogenic and/or non-pathogenic (Fo47) fungal strains. In this case, the modified method of Schuck et al., 2014 [39] was used. Young seedlings (21 day-old) were inoculated with the spores at a concentration of 10^6^ spores/mL prepared in sterile water with 0.1% Tween 20 solution, as described in Baghbani et al., 2018 [21]. Then, disease symptoms were assessed and the number of infected seedlings calculated 7, 8, and 11 days after inoculation according to the mycelium method described by Wróbel-Kwiatkowska et al., 2022 [40]. In this method, a 3-step scale is applied to assess the level of infection depending on the disease symptoms and necrosis. The numbers of infected flax plants were determined as a percentage of the total plants, which were monitored for 11 days. Finally, the degree of plant infection caused by pathogenic strains was evaluated.

### 3.6. Analysis of Content of Photosynthetically Active Pigments in Tested Flax Plants

Chlorophyll a and b and carotenoids were measured in plant tissues collected from in vitro cultures and after freeze-drying. The extracts obtained after extraction with 100% acetone were used for absorbance measurements using the Tecan microplate reader (Tecan Group Ltd., Männedorf, Switzerland) at wavelengths of 470 nm, 645 nm, and 662 nm. The analysis was performed as described by Lichtenthaler (1987) [41].

### 3.7. Determination of Total Polyphenol Content (TPC) and Total Flavonoid Content (TFC)

The total polyphenol content (TPC) was determined spectrophotometrically in tested lyophilized flax seedlings derived from in vitro cultures, following the Folin–Ciocâlteu method described by Makkar et al. (1995) [42] and Wróbel-Kwiatkowska et al. (2022) [40]. Folin–Ciocâlteu reagent was purchased in Merck, Germany. A portion (100 μL) of the final sample was transferred to the microplate wells, and the absorbance (at 750 nm) was analyzed using the Tecan microplate reader. The polyphenol content was calculated using a standard curve prepared with gallic acid (Merck, Darmstadt, Germany), and the results are expressed as milligrams of gallic acid equivalent (GAE eq.) per gram of plant dry weight.

The total flavonoid content (TFC) in lyophilized samples was determined using a colorimetric method with AlCl_3_ (Merck, Darmstadt, Germany), as described by Zhishen et al. (1999) [43] and Wróbel-Kwiatkowska et al. (2022) [40] with quercetin (Merck, Darmstadt, Germany), used as a standard. The absorbance of each sample was measured at 510 nm using the Tecan microplate reader. The results are expressed as milligrams of quercetin equivalents (QE eq.) per 100 g of plant dry weight.

### 3.8. Analysis of FTIR Spectra

FTIR/ATR spectra were recorded within the 4000–300 cm^−^^1^ range using a Nicolet 6700 spectrophotometer equipped with an ATR accessory (Thermo Fisher Scientific, Waltham, MA, USA). Each spectrum was acquired ten times and subsequently averaged. Spectral analysis was performed using commercial software (OriginPro 2025, OriginLab Corp., Northampton, MA, USA), which included background correction and deconvolution of the experimental bands into Lorentzian components. To enable a quantitative comparison of the individual components in the studied materials, the spectra were standardized. The reference point for the analyzed FTIR spectra was established as the band with maximum intensity at 2920 cm^−^^1^, corresponding to the ν(CH_3_) vibration. The integral intensities of the Lorentzian components were calculated relative to the integral intensity of this band. To evaluate the crystallinity index (ICr), which reflects the structural organization of cellulose, the ratio of the integral intensities of the bands at 1372 cm^−^^1^ (associated with in-plane bending vibrations of −CH_2_ and −CH_3_) and 2920 cm^−^^1^ (corresponding to the stretching vibration of −CH_3_) was determined.

### 3.9. Statistical Analysis

Data analysis was performed using Statistica 14 software (TIBCO Software Inc., Palo Alto, CA, USA) with a combination of parametric and non-parametric statistical methods. To meet the assumptions of normality and homogeneity of variance, data were first subjected to Box–Cox transformation prior to analysis. One-way analysis of variance (ANOVA) was then employed to detect significant differences among treatment groups, followed by the least significant difference (LSD) test for pairwise comparisons at a significance level of *p* < 0.05. For data that did not meet parametric assumptions, the Kruskal–Wallis test was used as a non-parametric alternative, supplemented with Dunn’s post hoc multiple comparisons with Bonferroni correction to determine significant differences among groups. The degree of plant infection, expressed as categorical frequency data, was assessed using the Chi^2^ test of independence, and standardized residual heatmaps were generated to highlight significant deviations between observed and expected distributions. All statistical analyses were conducted using a significance threshold of *p* < 0.05.

## 4. Conclusions

This study highlights the strong potential of non-pathogenic fungi (such as the tested Fo47) as protective biocontrol agents for plants against fungal infections (especially for *R. solani* and *F. culmorum* attack). The mechanism of non-pathogenic fungal action in plants potentially involves the induction of secondary metabolite synthesis (total flavonoids and total phenolic compounds) and biochemical and structural modifications of the plant cell wall, resulting in improvement in plant resistance to pathogens. These results are important from a research and application point of view. They suggest the role of non-pathogenic fungal strains in plant resistance and their potential in plant protection against pathogens. This is especially important nowadays, as new methods are needed to replace fungicides and because of the growing problem of fungicide-resistant fungal strains [44]. Further analysis on flax plants grown in field conditions protected with the Fo47 strain and infected with pathogenic fungi will be carried out in the near future, as well as analysis of mechanisms involved in induced systemic resistance (ISR). In this case, plant gene expression will be analyzed to check which genes/pathways are engaged in this type of resistance triggered by biocontrol non-pathogenic fungi.

## Figures and Tables

**Table 1:** Comparison of integral intensities of selected characteristic bands for cellulose identified for flax plants infected with *F. culmorum* (Fc) or *F. culmorum* and simultaneously with non-pathogenic strain Fo47 (Fo47 + Fc). Flax plants not infected with any fungi served as a control (C).

Wavenumber (cm^−1^)	Assignments	Relationship
1450	δ(CH) + ω(CH_2_) + δ(OH)	Fc ≤ C < Fo47 + Fc
1410	δ(CH) + ν(φ) + δ(OH)	C ≤ Fo47 + Fc < Fc
1315	δ(CH) + δ(OH)	C ≤ Fc < Fo47 + Fc
898	ν(φ) + ρ(CH_2_)	C ≤ Fo47 + Fc ≤ Fc
1150	ν(C-O-C)	C ≈ Fo47 + Fc < Fc

**Table 2:** Comparison of integral intensities of selected characteristic bands for lignin identified for flax plants. C (control, flax not infected with any fungi), Fc—flax plants infected with *F. culmorum*. Fo47 + Fc—flax plants treated with strain Fo47 and infected with *F. culmorum*.

Wavenumber (cm^−1^)	Assignments	Relationship
1545	ν(C=C)	C ≤ Fc < Fo47 + Fc
1508	ν(φ)	C ≤ Fc < Fo47 + Fc
1336	ν(C-O) + ν(φ)	C < Fc < Fo47 + Fc
826	δ(φ) + δ(CH)	C ≤ Fc < Fo47 + Fc

**Table 3:** Relationship of integral intensities of selected characteristic bands for pectin identified for flax treated with Fo47 and infected pathogenic strain *F. culmorum* (Fo47 + Fc) and for flax infected only with *F. culmorum* (Fc). Flax plants not treated with any fungi served as a control (C).

Wavenumber (cm^−1^)	Assignments	Relationship
1740	ester carbonyl groups ν(C=O)	C ≈ Fc < Fo47 + Fc
1705	ester carbonyl groups ν(C=O)	Fc ≤ C < Fo47 + Fc
1647	free or ionized carboxyl groups ν_as_(COO^−^)	C ≤ Fc < Fo47 + Fc
1600	free or ionized carboxyl groups ν_s_(COO^−^)	Fc ≤ C < Fo47 + Fc

## Data Availability

Data are contained within the article.

## Supplementary Materials

The following supporting information can be downloaded at: https://www.mdpi.com/article/10.3390/ijms26094396/s1.
