# Supplementary material for: The Influence of the Non-Pathogenic Fusarium oxysporum Fo47 Strain on Flax Resistance to Pathogens
Source: Int J Mol Sci. 2025 May 6;26(9):4396. doi: 10.3390/ijms26094396 (PMC12072775; doi:10.3390/ijms26094396)
Supplement: Supplementary file 1 [file ijms-26-04396-s001.zip › ijms-3591818-supplementary.pdf]

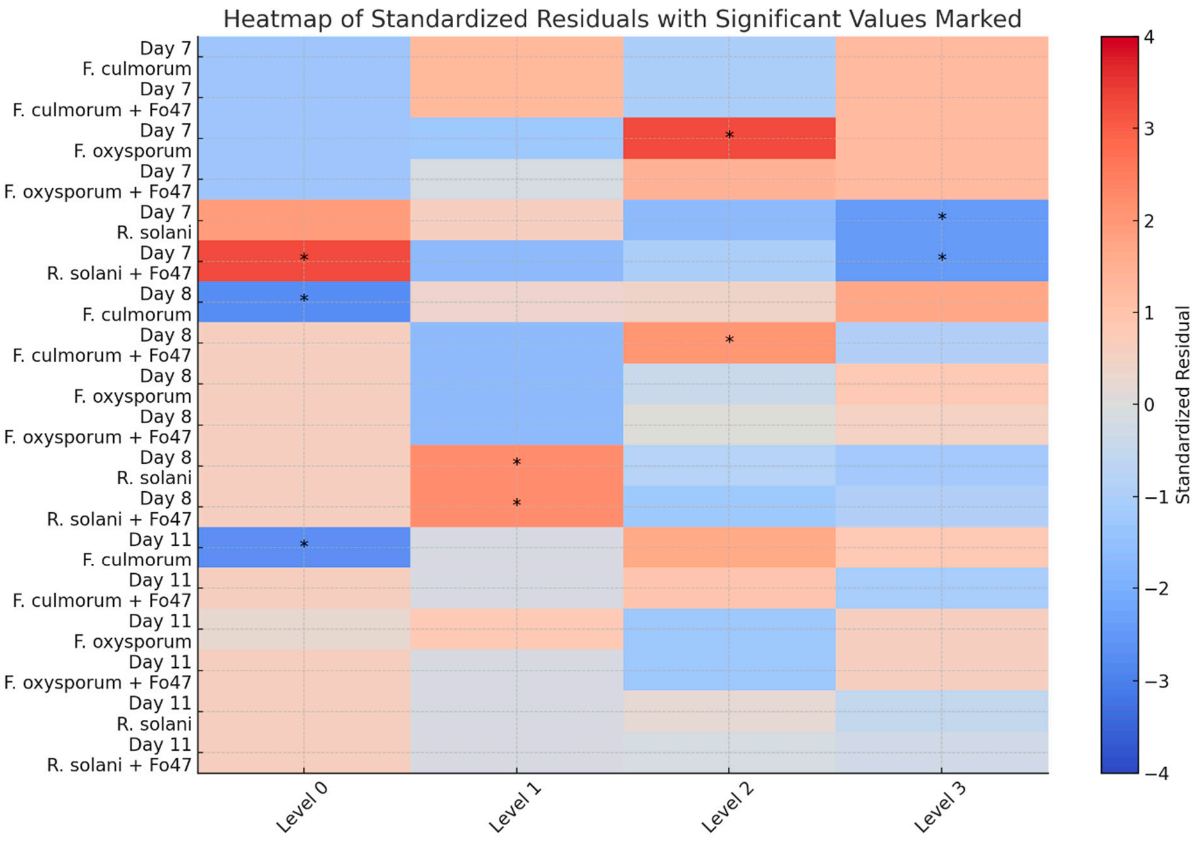

Figure S1: Heatmap of standardized residuals illustrating the association between treatment groups of **Jan cultivar** and infection levels across experimental days. Residuals greater than |2|, indicating statistically significant deviations from expected frequencies, are marked with an asterisk (*). Positive residuals represent overrepresentation, while negative residuals represent underrepresentation compared to the expected distribution under the null hypothesis of independence.

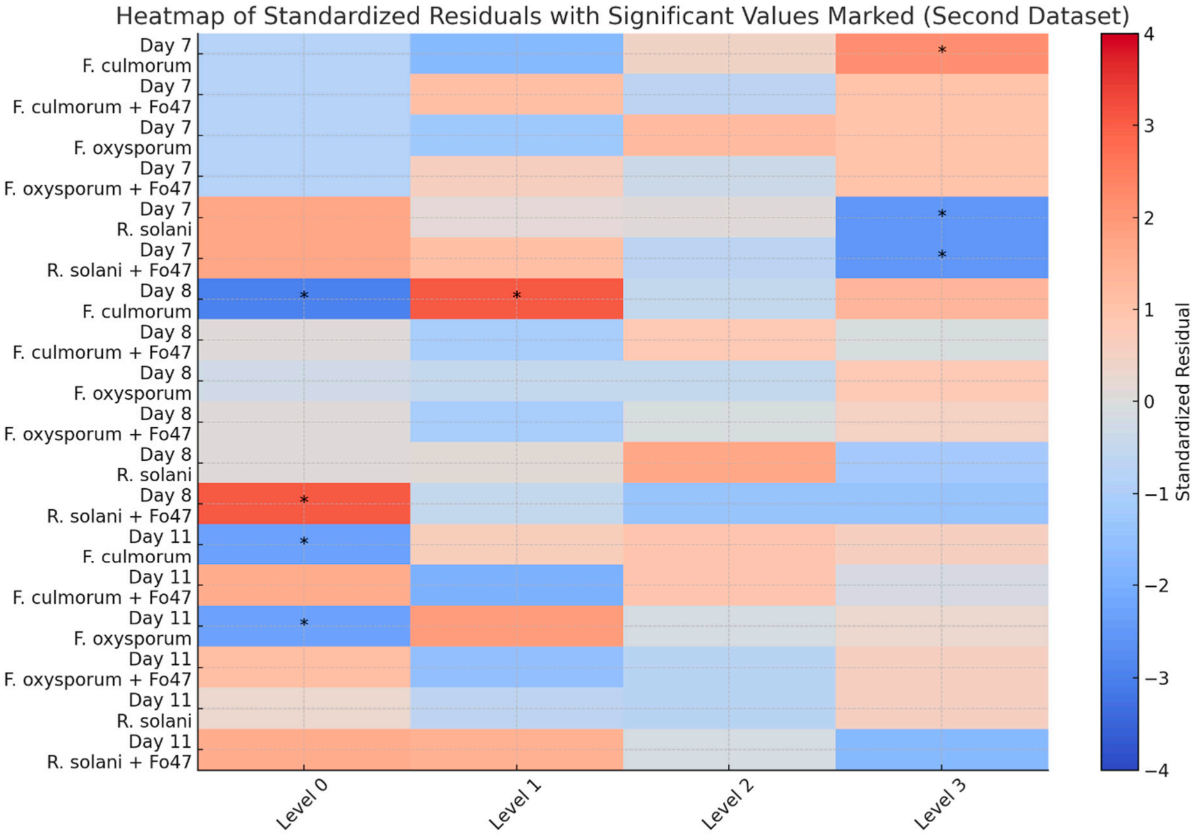

Figure S2: Heatmap of standardized residuals illustrating the association between treatment groups of **Bukoz cultivar** and infection levels across experimental days (second dataset). Residuals exceeding |2|, indicating statistically significant deviations from expected frequencies, are marked with an asterisk (*). Positive residuals represent overrepresentation, while negative residuals represent underrepresentation compared to the expected distribution under the null hypothesis of independence.
